# The Road to Unconventional Detections: Paper-Based Microfluidic Chips

**DOI:** 10.3390/mi13111835

**Published:** 2022-10-27

**Authors:** Yuhang Jin, Aziz ur Rehman Aziz, Bin Wu, Ying Lv, Hangyu Zhang, Na Li, Bo Liu, Zhengyao Zhang

**Affiliations:** 1 Liaoning Key Laboratory of Integrated Circuit and Biomedical Electronic System, School of Biomedical Engineering, Faculty of Medicine, Dalian University of Technology, Dalian 116024, China; 2 School of Life Science and Pharmacy, Dalian University of Technology, Dalian 116024, China; 3 China Certification and Inspection Group Liaoning Co., Ltd., Dalian 116039, China

**Keywords:** paper-based microfluidics chips, biomedicine, biomarker, clinical detection

## Abstract

Conventional detectors are mostly made up of complicated structures that are hard to use. A paper-based microfluidic chip, however, combines the advantages of being small, efficient, easy to process, and environmentally friendly. The paper-based microfluidic chips for biomedical applications focus on efficiency, accuracy, integration, and innovation. Therefore, continuous progress is observed in the transition from single-channel detection to multi-channel detection and in the shift from qualitative detection to quantitative detection. These developments improved the efficiency and accuracy of single-cell substance detection. Paper-based microfluidic chips can provide insight into a variety of fields, including biomedicine and other related fields. This review looks at how paper-based microfluidic chips are prepared, analyzed, and used to help with both biomedical development and functional integration, ideally at the same time.

## 1. Introduction

The perfect integration of medicine and engineering has led to the development of many biotechnologies, such as marker detection, gene amplification, cell culture, etc. In professional laboratories, however, professional instruments are used, which raises costs, makes relevant experiments more difficult, and restricts discipline development. So, biomedical research has slowly moved toward making new devices that are economical, easy to use, environmentally friendly, and yield great results.

In 1990, microfluidics was first proposed for microfluidic control in microelectromechanical systems (MEMS) [1]. Microfluidic chips were initially applied for chemical analysis [2]. Among different types of microfluidic chips, paper-based microfluidic chips have recently gained attention in the biomedical field because of their potential for developing into ideal biomedical devices [3,4].

This review examines key developments in paper-based microfluidic chips from three perspectives: channel preparation methods, analytical techniques, and their current applications. Moreover, we have discussed some new opportunities for innovations that could move us beyond the current state of the art. These opportunities could enable these chips to be used for societal purposes.

## 2. Overview of Paper-Based Microfluidic Chips

### 2.1. Characteristics of Paper-Based Microfluidic Chips

Microfluidic chips are small and lightweight devices with highly integrated detection. It integrates sample preparation, reaction, separation, detection, and other components at the micron scale level with fine processing technology. As a result, it can carry out complex physical and chemical processes as well as complete the whole experimental analysis, from adding samples to reading results. Therefore, it is also called “lab on chip” [5,6,7]. Whitesides’ research group at Harvard University in the United States proposed the concept of paper-based microfluidic analytical devices (μPADs) in 2007 [8]. For “lab on papers”, filter paper is used as a substrate for microfluidic chips instead of inorganic or polymeric materials. There are several advantages to using paper-based microfluidic chips: the paper itself has a capillary effect, which can guide reagent flow without requiring additional power sources. Paper is relatively inexpensive, easy to obtain and process, so chip preparation cost is reduced. Similarly, the size is small and the volume is light, making it easy to transport and store. As it degrades more easily than other synthetic materials, it reduces the environmental restrictions on chips’ use and is more environmentally friendly. Thus, μPADs are more economical, safer, and easier to use and transport than other microfluidic chips [9,10,11].

### 2.2. Materials of Paper-Based Microfluidic Chips

The design and preparation of a paper-based material plays a decisive role in determining how well the μPADs will perform. There are currently three paper-based materials widely used for the production of paper chips: filter paper [12], nitrocellulose paper [13], and glass fiber paper [14]. All of them have their own characteristics and applications (described in Table 1), and can also be used alone or combined with each other to achieve the overall function. Furthermore, parameters such as the thickness of the paper and the porosity of the paper also affect fluid velocity. These parameters should be taken into consideration when performing more detailed fluid control calculations [15].

## 3. Preparation Method of Paper-Based Microfluidic Chip

### 3.1. Two-Dimensional Paper Chip Preparation

Paper-based chips differ from glass, plastic, or other materials-based chips. For example, the hydrophilic channels flanked by hydrophobic barriers in paper-based microfluidic devices guide liquid flow and the reaction is etched on the substrate. As technology develops, paper chip preparation methods become more sophisticated. Paper in a broad sense includes various analytical devices prepared by simple splicing or stacking of paper-derived materials. Fluid control is undoubtedly a top priority in paper chip design [16]. This section focuses on common methods for making channels on paper for fluid flow (as described in Table 2) [17,18,19].

#### 3.1.1. Photolithography

**Figure 1:**
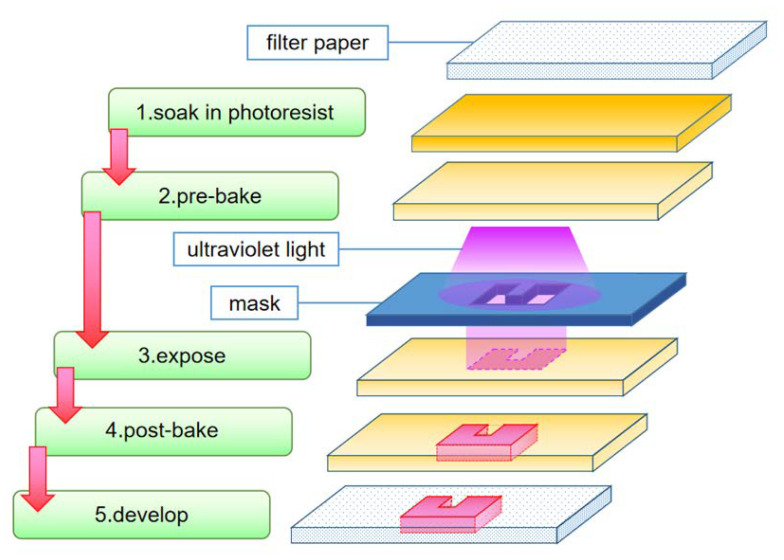
Schematic diagram of paper chip preparation using photoresist.

In the field of microelectronics, photolithography is used to prepare chips. Through the crosslinking reaction of the photoresist and exposure to ultraviolet light, the substrate becomes resistant to corrosion, and the pattern on the mask is transferred to the substrate (Figure 1).

In the Whitesides group, paper chips are obtained through the same photolithography method [8]. The filter paper is first soaked in the SU-8 photoresist and heated to dry. Then, it is exposed to UV light through a hollowed-out mask with a specific shape plate coating. The hollowed-out part of the template can form a hydrophobic structure. The filter paper is then immersed in propylene glycol methyl ether acetate to remove excess photoresist. Finally, it is exposed to oxygen plasma to enhance its hydrophobicity so that a paper chip with a specific channel structure is produced. Similarly, using photolithography technology, Busa et al. [20] developed hydrophobic barriers on various paper substrates to separate hydrophilic areas and compared them in year 2016. They concluded that “cellulose membrane has better stability in photolithography than nitrocellulose membrane”. Accordingly, a simple and portable paper chip for horseradish peroxidase detection was designed and manufactured. The preparation of paper chips using photolithography is cumbersome, and the soaking and drying process of photoresist will destroy the toughness of the paper substrate, making them unsuitable for folding. In spite of this, paper chips are still prepared using this method, but its practical application is very limited.

#### 3.1.2. Plasma Treatment Technology

Upon repeated ionization, the material becomes a plasma state, exhibiting suitable flow, diffusion, and gas-like properties. Paper chips can also be prepared using plasma treatment technology, which uses high-energy plasma impacts to change the chemical structure of polymers and modify the surface of materials. Li et al. [21] soaked the filter paper sample in alkyl ketene dimmer (AKD) solution (0.6 g/L), and then heated it up to 100 °C for 45 min to make it hydrophobic. After clamping the filter paper between two metal plates with a specific hollow shape, it was subjected to plasma treatment to restore its hydrophilicity. According to Hecht et al. [22] plasma etching and plasma polymerization were two methods of preparing paper chips in which makes the substrate selectively hydrophobic and hydrophilic, respectively. For the preparation of paper chips, plasma etching is more efficient and cheaper, making it more suitable to use. These methods are low cost and do not destroy the flexibility of the filter paper. The disadvantage is that the preparation is less flexible due to the dependence on the template.

#### 3.1.3. Wax Printing

**Figure 2:**
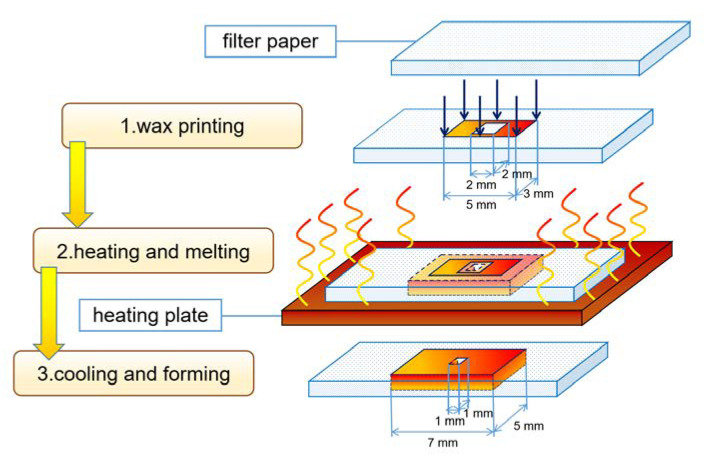
Schematic diagram of paper chip prepared by wax printing.

In wax printing, paraffin is attached to paper-based materials in a prescribed shape, then heated to melt the paraffin and allow it to penetrate the paper to form a hydrophobic barrier so that a channel is created (Figure 2). Zhong et al. [41] compared the sample transport functions of A4 printing paper, napkins, and channels with different waxes and concluded that the experimental paper has the best sample transport function. Using the wax printing method, Lu et al. [25] explored the specific conditions for heating the paraffin on the surface of the filter paper: when the heating temperature was 110 °C, it took 5 min for the paraffin to penetrate the filter paper completely, whereas when the temperature was 130 °C, it took only 30 s to achieve the same effect. Martins et al. [24] fabricated a paper-based electrochemical sensor for the detection of 3-nitrotyrosine (3-NT) by printing a well-designed hydrophobic barrier on cut paper using a wax jet printer. Although the paraffin could diffuse horizontally after heating, the degree of diffusion is limited, and the effect can be reduced by reserving space for the wax to diffuse. A millimeter chip can be prepared using this method without chemical reagents, which makes it easy to prepare, safe to use, and widely applicable.

#### 3.1.4. Inkjet Method

Similar to the wax printing method, the paper chip channels are manufactured by printing specific hydrophobic ink on the filter paper using an inkjet printer. For instance, Li et al. [42] printed the hydrophobic reagent (2% solution of octadecyl trimethoxysilane in N-heptane) on filter paper and heated the filter paper at 100 °C for 90 min using a heating plate, then cooled at room temperature for 3 h. Finally, the filter paper was treated with an oxygen plasma cleaner for 4 min to prepare inkjet-printed paper chips. By directly printing the AKD solution onto the paper base, Shen et al. [27] created a hydrophobic area without heating it. Moreover, Hamidon et al. [43] investigated an AKD ink with better device compatibility and material penetration. Hydrophobic ink does not spread by heat such as paraffin because it works on a different principle. Therefore, the channels drawn by the inkjet method are more accurate than the wax printing method.

Graphic design is performed on computers for both wax printing and inkjet printing, so specialized printing equipment is needed for efficient production. The high cost is one of the disadvantages of these devices. Recently, wax jet printers have been widely used in laboratories, leading to significant research on them. The hydrophobic ink used in inkjet is a liquid that has the characteristics of regular ink and can be loaded into the water-based pen to draw channels, thus eliminating the need for inkjet printing equipment. However, manual drawing inevitably brings the problem of precision. Additionally, hydrophobic inks such as siloxane can be printed with home-type printers, which solves the problems of production cost and preparation accuracy.

#### 3.1.5. Screen Printing

**Figure 3:**
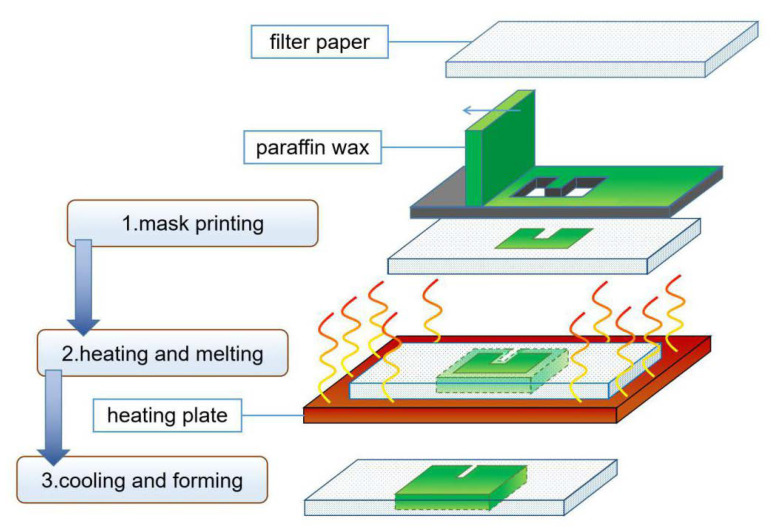
Schematic diagram of paper chip prepared by screen-printing method.

Screen-printing technology is the process of injecting ink into the printing plate and applying pressure to print the pattern on the substrate, such as a screen-printing plate with graphics and text on it (Figure 3). Dunchai et al. [31] used screen-printing technology to print paraffin on the surface of filter paper in a specific pattern. By heating and cooling paraffin to room temperature, a hydrophobic channel wall structure can be constructed. This method does not require complicated and expensive equipment. It is suitable for low-cost production but needs different screen-printing plates for different chips, which limits its flexibility.

#### 3.1.6. Laser Processing Technology

CO_2_ laser etching is used in laser processing to create a hydrophilic channel with a specific shape and depth on a hydrophobic substrate. In order to increase the hydrophilicity of these channels, nano-SiO_2_ particles can be applied inside them. Mahmud et al. [32] lined the lower layer of filter paper with aluminum foil. Laser was used to cut the filter paper with the hydrophobic barrier to prepare a paper chip. This method produces high precision paper chips, but expensive laser equipment makes it difficult to popularize, despite the theory of making such chips already being discussed.

### 3.2. Three-Dimensional Paper Chips Preparation

In fact, three-dimensional paper chips are just a superposition of two-dimensional paper chips, but they achieve the goal of “1 + 1 > 2”. By providing a multilayer structure and vertical flow channels, the flux of the paper chip is increased and the layers of the paper chip are enriched, allowing for a more controlled detection reaction in time and space, one of the new directions for paper chip development [44,45]. In comparison to 2D paper chips, 3D paper chips are more difficult to fabricate at a higher cost, but they are more convenient, accurate, and sensitive [46].

#### 3.2.1. Origami Method

The origami method is a way to make a three-dimensional paper chip by cutting a piece of paper into different parts and making channels with different shapes for each part. The paper is then folded in a certain order. Cai et al. [35] made a paper chip with specific hydrophilic and hydrophobic structures. The chip was split into two equal parts, yellow and green, by heating the filter paper at 120 °C for 5 min after printing wax on it with a wax spray printer. Then, a Taqman probe and double-strand specific nuclease (DSN) were added to the hydrophilic regions of the yellow and green parts, respectively. The two parts were folded after the reagents were dried at room temperature (25 °C). For instance, a paper-based microfluidic chip based on the DSN amplification principle was fabricated for detecting cancer biomarkers microRNA (miRNA). Since the paper core has only two layers, it achieved structural stability without fixing. Xiao et al. [36] folded filter paper into five layers and fixed them with a phoenix clip to prepare a highly sensitive silver ion detection sensor. Although the origami paper chip has obtained better performance, the layers of the three-dimensional paper chip need to be fixed by reasonable means. Thus, the origami method is simple and easy to prepare. It takes the structural design of multilayer channels as its core. In addition, the fixing method can ensure a close connection between layers. While the smooth channel is also very important, it may break the continuity of vertical hydrophilic channels compared with double-sided adhesive tape. Clips and return pins can avoid this trouble with a little increasing cost.

#### 3.2.2. Lamination Method

**Figure 4:**
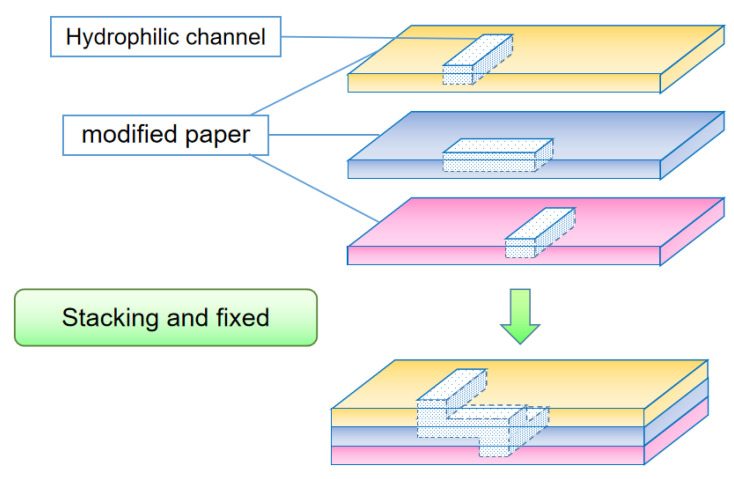
Schematic diagram of 3D paper chip prepared by lamination method.

Unlike the origami method, which prepares three-dimensional paper chips on the same piece of paper, the lamination method prepares different levels of channel structures on multiple sheets of paper with the same shape and size. Then, a double-sided tape, clip, or any other device is used to fix them into a whole paper chip (Figure 4). Wang et al. [47] constructed a paper-based microfluidic analysis device composed of four layers of different structures superimposed on each other. They made a hydrophobic barrier on the filter paper by the wax printing method and prepared a colorimetric analysis system for carcinoembryonic antigen. Hao et al. [38] used a wax jet printer to print out the hydrophobic barrier and hydrophilic channel structures on the paper. Three printed papers were assembled top to bottom to produce a general fast ratio fluorescence sensing platform for paper-based microfluidic chips; the top layer with a guiding fluid channel, a middle layer with a reagent-treated polyester fiber membrane, and a bottom layer capable of storing liquid. The lamination method is especially suitable for three-dimensional paper chips with different materials in each layer. Since there is not a clear fold line to fix the relative positions of theses layers, there is also the problem of how to line up the channel structure of each layer.

#### 3.2.3. Other Methods

Researchers are trying to find new methods to prepare three-dimensional paper chips better than the classic origami and lamination methods. Jeong [39] et al. printed different patterns by spraying wax on both sides of the filter paper. Then, the paraffin on both sides was heated precisely so that it penetrated to an ideal depth and connected to each other in three-dimensional channels. The pinwheel-shaped 3D paper chip designed and prepared by Wang et al. [40] is fixed by a plastic shell. Through the process of rotation, four kinds of heavy metal ions (Cu^2+^, Cd^2+^, Pb^2+^, and Hg^2+^) can be sensitively detected at one time. Due to the high cost and difficult operation, the application of these methods in the preparation of 3D paper chips is relatively rare, but they help 3D paper chips achieve multiple functions, propose unprecedented innovations, and have broad potential for development.

## 4. Analysis Method of Paper-Based Microfluidic Chip

The paper-based microfluidic chip focuses on the results being presented in a visual way. Using colorimetry, electrochemistry, and fluorescence, the paper chip can find out both qualitative and quantitative information about different substances based on their physical, biological, and chemical properties [48,49,50] (described in Table 3).

### 4.1. Colorimetric Method

The chromogenic method is a well-known way to observe the results by looking at the change of color of a certain area before and after the reaction. This method uses the physical deposition of colored substances to make the color. These colored substances are often used to mark the reactants to obtain a visualization of the reaction result, which would not produce color change by itself. The lateral flow analysis is based on the chromogenic method. In contrast, colloidal gold is a classic reagent used for chromometric detections [62,63]. For instance, the well-known lateral flow analysis detection device “pregnancy test paper” employs colloidal gold immunochromatography for human chorionic gonadotropin detection [51]. By using an antibody fixed on a nitrocellulose membrane, it deposits a red gold-labeled antibody complex and shows red strips that can be recognized by the naked eye for detection of results [64,65]. In 2007, Gao’s research team [66] found that Fe_3_O_4_ nanoparticles are similar to colloidal gold particles, whose peroxidase properties can cascade and amplify the detection signal through enzyme-catalyzed reaction. The sensitivity of immunochromatography based on Fe_3_O_4_ is about 100 times higher than that of colloidal gold [67]; thus, work on Fe_3_O_4_ nanoparticles is becoming one of the new landscapes of chromogenic paper chips.

**Figure 5:**
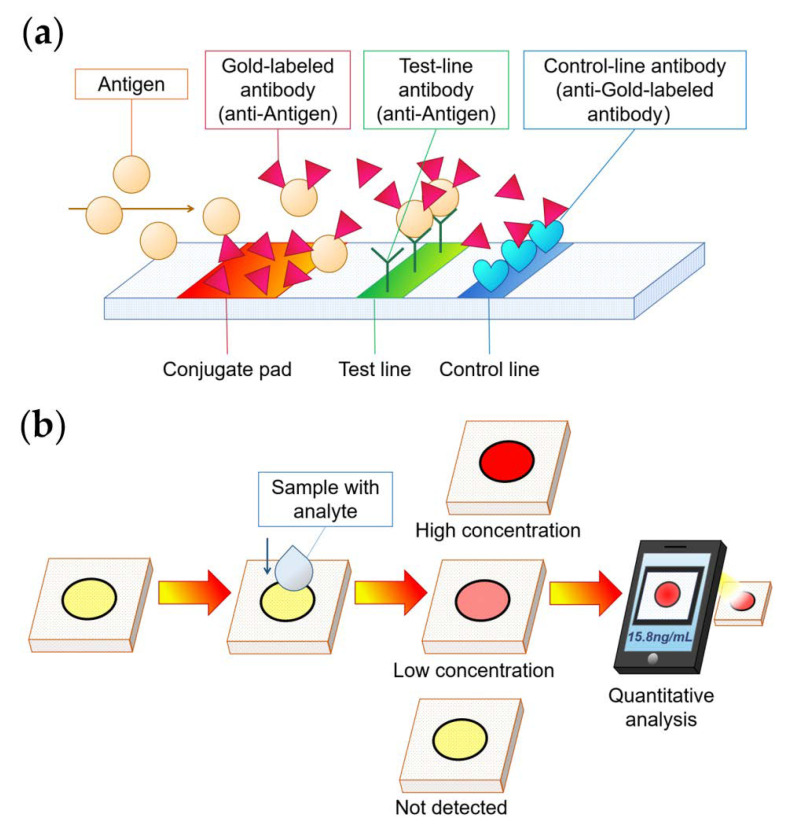
Paper chip for marker detection by colorimetry. (**a**) Chromogenic method in lateral flow analysis to achieve colloidal gold immunochromatography [64]; (**b**) the principle of color-changing methods of the analyte [72].

In contrast to chromogenic methods based on physics, the methods based on chemical reactions to produce color changes are color-changing methods. Hossain et al. [68] developed a multi-channel heavy metal detection paper chip with suitable sensitivity. The chip’s functioning depends on the property of heavy metal ions so that they can change the activity of β-galactosidase in conjunction with the color reaction of other metal ions. Based on the chemical properties of benzoic acid, Liu et al. [69] developed a paper chip that can quantitatively detect the concentration of benzoic acid in food by analyzing color development through a smartphone program. Wei et al. [52] prepared the paper chip using urea/phenol red impregnation technology. The chip employed an H_2_O_2_-treated Fe_3_O_4_@Ag multifunctional hybrid nanoprobe (APT-Fe_3_O_4_@AgNP) solution to generate Ag^+^. The Ag^+^ in the supernatant inhibited urease activity. As a result, when salmonella typhimurium was specifically captured in the solution, the bacteria were able to adsorb Ag^+^ in the supernatant. So, the supernatant no longer inhibited urease activity on paper, which caused urease to catalyze urea into a large amount of [NH₄]^+^. Briefly, [NH₄]^+^ reacts with the reagents on the paper chip to produce a significant pink color change for rapid and sensitive colorimetric detection. Since paper chip substrates are usually white, qualitative test results are easily readable by the naked eye. It is supposed to use smartphones, digital cameras, and other equipment [53]. Some image processing software can also be used for the quantitative analysis of parameters such as grayscale [70] and color rendering area [71]. In particular, colorimetry is a classic and commonly used for paper-based microfluidic chips for the detection of different substances (shown in Figure 5).

### 4.2. Electrochemical Method

Therefore, it measures the potential change through electrodes mounted on paper sheets, which are usually made of special inks such as Ag^+^/AgCl inks, carbon black, and more recently developed boron-doped diamond (BDD) electrodes printed [73,74,75] or metal sputtered [56] (mainly gold electrodes). Jemmeli et al. [54] used carbon black, a nano material, to print electrodes on the filter paper to prepare a sensor with high sensitivity but low cost, which can detect bisphenol A (BPA) in water by adding samples only. Yang et al. [55] used screen-printed electrodes to measure the impedance change caused by enzyme inhibition reaction on a five-layered paper chip, and successfully distinguished three different pesticides. Recently, some researchers combined nanoparticles with electrochemical paper chips to increase detection sensitivity. In a study, Zhou et al., introduced CuCo-CeO_2_ nano-spheres that are spherical in shape and have a particle size of 200 nm, a rough surface, and can effectively combine with DNA [76]. As a metal oxide nanomaterial with mixed surface valence, its unique electrocatalytic performance can enhance the electrochemical signal, providing ultra-sensitive detection of miRNA-155 in serum samples. However, electrochemical detection needs the support of professional equipment such as an electrochemical workstation. Recent research has designed a paper chip system that can realize self-power generation with the help of capacitance and read the results only with a multimeter [77]. Moreover, these chips can achieve accurate quantification of results within their sensitive range. However, the use of electrochemical paper chips is still a relatively complex process and needs further research in this field.

### 4.3. Fluorescence Method

Fluorophores absorb energy in specific wavelength areas, then reemit it in other wavelength areas. This causes fluorescence of different colors to be produced to visualize the results of the analysis. For instance, the chemiluminescence method uses chemical reactions to produce luminescence, while the electrochemical luminescence method uses voltage to drive the chemical reaction. It includes the chemiluminescence method, which makes the reagent glow through a chemical reaction, and the electrochemical luminescence method, which applies voltage to make the chemical reaction proceed. Ali et al. [59] prepared a new three-peak system based on paper-based microfluidic devices, which can help police grab ketamine as long as samples are added to the detection area. Delaney et al. [57] used the electrochemiluminescence method to screen-print the electrode on the paper chip with inkjet-printed channels and then applied voltage to achieve quantitative detection of 2-(dibutylamino) ethanol (DBAE) and nicotinamide adenine dinucleotide (NADH) based on the luminous intensity. Recently, Ezequiel’s team [78] optimized electrochemiluminescence to develop a single-electrode electrochemical detection paper chip using dye contained in commercial glow sticks with carbon paint electrodes. The system not only reduced detection cost but also increased detection flexibility by detecting H_2_O_2_. The advantage of the fluorescence method is that it is more sensitive. This is because even very weak fluorescence can be seen with fluorescence detection equipment, such as a fluorescence microscope, which can detect targets at micro-scale levels. However, fluorescence detection equipment must be used to read the results, which can be easily affected by the fluorescence emitted from the basic background of the paper.

### 4.4. Combining with Electronic Equipment

Accurate analysis results often require professional equipment and laboratory professionals. However, research has gradually improved paper chip analysis and detection capabilities, resulting in more accurate analysis results. The popularity of smart phones has led some researchers to develop paper chips that can be used with mobile phones. They can achieve sensitive insights into fluorescence intensity [58] or precise color-grayscale analysis [4,61,79] with mobile phone cameras and apps such as ImageJ, etc. Furthermore, some electrochemical workstations are gradually becoming home testing equipment, such as blood glucose meters [60], which have gradually reduced in size and become more portable. Carbon-13 labeling is also being used in hospitals for the detection of Helicobacter pylori breath. In this method, subjects blow air into the paper chip to determine the number of colonies in the body. They insert it into the electrochemical workstation, which not only greatly reduces the cost of detection but also avoids the physical trauma caused by traditional gastroscopic sampling methods. The combination with electronic equipment may reduce the lightweight advantage of paper chips, but medical equipment development and popularization could lead to better development prospects.

## 5. Application of Paper-Based Microfluidic Chip

### 5.1. Biochemical Marker Detection

The most classic and common use of paper-based microfluidic chips is to detect various biochemical markers in fields of food safety [80,81], environmental protection [82], and medical health [83,84], especially the detection of disease-related markers [85,86]. Using the above-mentioned methods, the paper chip can be prepared to detect various biochemical markers such as inorganic substances [71], proteins [87], and nucleic acids [88,89,90]. Ye et al. [91] combined paper-based devices with smart phones to enhance the sensitivity of quantitative detection of the Hg^2+^ ions in water samples. Boonkaew et al. [92] used the wax printing method to prepare paper chips and screen printing to obtain printed graphite electrodes for the electrochemical detection of C-reactive protein in human serum samples. Noviana et al. [93] prepared a paper chip based on a nuclease protection assay. The change in color of the reaction area can be used to find the target nucleic acid. Li et al. [94] developed a fluorescent paper-based sensor that can detect folate in solution.

From one to two channels and then to more, it is a leap from quantitative change to qualitative change. For more accurate results, many analyses and tests need to consider multiple markers, especially in disease detection. It is becoming increasingly critical to research and design multiplexing, which significantly improves the detection accuracy and efficiency of detection. This is performed through the preparation of additional channel structures and the addition of hydrophobic materials between the existing layers of channel structures [74,95,96].

Besides the detection of collected samples in the laboratory, the lightweight and compact characteristics of paper chips make them suitable for being used as wearable sensors for non-invasive monitoring of human health in daily life. Xiao et al. [97] and Castro et al. [98] developed wearable colorimetric sensors that can detect glucose in sweat and saliva, respectively. So, these sensors can monitor the physical condition of users at any time. The exploration of outdoor equipment has always been a hot topic for inventors. Even though facing more difficulties, it needs to be further developed and improved.

### 5.2. Nucleic Acid Preparation

#### 5.2.1. DNA Extraction

**Figure 6:**
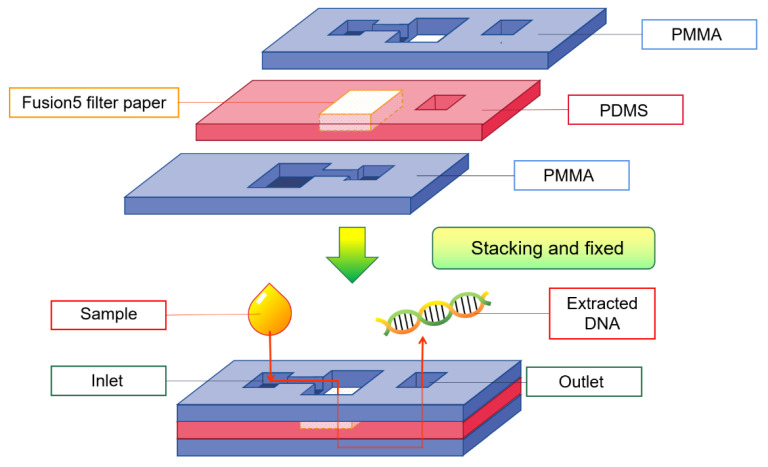
DNA extraction device designed by Gan et al. [99].

The extraction of target DNA is particularly important in a variety of scientific research fields. However, the conventional method of extracting DNA requires complex processes such as destroying cells, centrifuging and layering, heating, or adding reagents. These processes rely on professional equipment and must be carried out in the laboratory. Gan et al. [99] assembled the filter paper in the middle of the plastic shell to make a DNA extraction paper-based apparatus (shown in Figure 6). They successfully extracted biological DNA from the original samples, including dried blood stains and cigarette butts. Through primer-mediated and enzymatic catalysis, the obtained DNA can be amplified by polymerase chain reaction (PCR) for subsequent detection, which operates on the principle of DNA semiconservative replication to exponentially amplify target DNA. In 2017, Tang et al. [100] prepared a paper-based device with sponge storage modules and paper valves for the rapid extraction of DNA from biological samples. It takes only 2 min to obtain target DNA from samples such as serum, saliva, and bacterial suspension. It has great potential to complete the whole process of DNA extraction outside the laboratory and even in the absence of specific conditions.

#### 5.2.2. Nucleic Acid Amplification

The amount of nucleic acid directly extracted from various biological samples is usually only trace amounts, which makes it difficult to reach the level used for detection. So, specific DNA fragments need to be amplified. PCR is the most common and typical method for DNA amplification in the lab. Conventional PCR requires sample processing, and the amplified DNA also needs to be detected by gel electrophoresis. Patil-Joshi et al. [101] used filter paper as the carrier of PCR samples, eliminating the need for sample processing. They not only successfully achieved the amplification of target DNA but also improved the efficiency of DNA replication. This method can be used for many different samples, and it makes the experimentation process much easier. The paper chip prepared by Chen et al. [102] integrates nucleic acid extraction, amplification, and detection functions. It can identify the EGFR mutations in lung cancer cells within 90 min and carry out the sensitive detection of lung adenocarcinoma, which is very powerful and worth popularizing.

In recent years, thermostatic amplification technology based on recombinase polymerase amplification (RPA) and recombinase-mediated amplification (RAA) has developed rapidly. Using a specific recombinant enzyme, the amplification of the target DNA can be achieved at 37–40 °C in 10 to 30 min. Therefore, it can cooperate with the paper chip. Colloidal gold immunochromatography can be used to find the target DNA by adding only a probe with a FAM fluorescent group to the amplification process and using an antibody against FAM. Heeseop et al. [103] used a paper chip made of glass fiber paper to carry out the RPA process on it. They performed simultaneous detection of a variety of foodborne pathogens by fluorescence method. Therefore, the combination of thermostatic amplification technology and paper chips is becoming the latest hotspot for nucleic acid detection. In addition, the latest research shows that the reverse transcription of mRNA samples can also be performed on paper-based equipment [104], further expanding the application of paper-based microfluidic chips in the field of nucleic acid detection.

### 5.3. Cell Analysis

**Figure 7:**
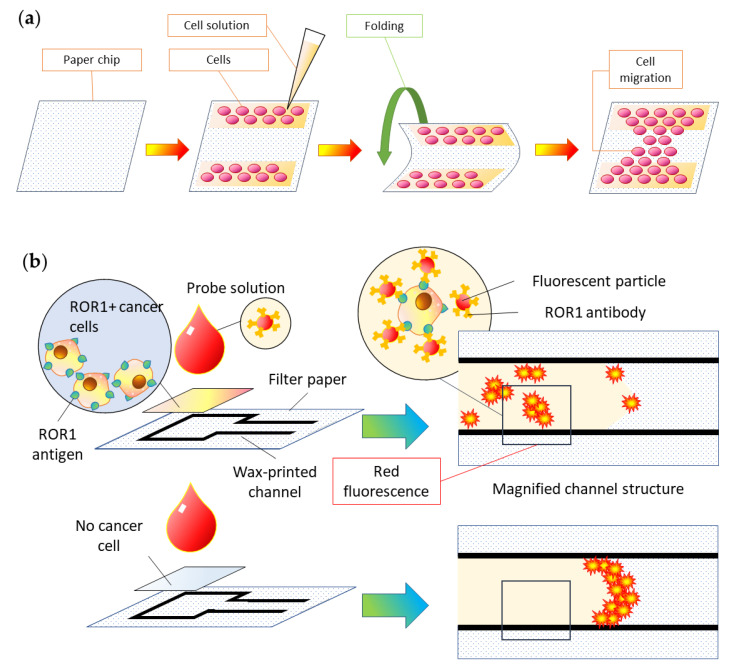
Paper chip-based cell analysis. (**a**) Cell stress stimulation device developed by Kaarj et al. [105]; (**b**) ROR1+ cancer cell capture paper chip developed by Ulep et al. [58].

In addition to analyzing and detecting biochemical markers at the molecular level, paper-based microfluidic chips can also be used for cell-level research, including cell separation, culture, stimulation, detection, and so on. The paper-based in vitro tissue chip developed by Kaarj et al. [105] can be knocked and bent through a program to provide local compression and shear flow mechanical stimulation to rat vascular endothelial cells fixed on it. As a result, it could provide insight into the specific impact of mechanical stimulation on angiogenesis as well as the possibility of using it for the treatment of malignant tumors that inhibit the growth of blood vessels (shown in Figure 7a). Ulep et al. [58] conjugated anti-ROR1 (receptor tyrosine-like orphan receptor one) and ethanolamine to highly carboxylated red fluorescent polystyrene particles having a diameter of 1 μm, forming a double-layer paper chip that can directly identify ROR1+ cancer cells from blood samples. Even ROR1+ cancer cells can be captured by observing the red fluorescence intensity with a special fluorescence microscope based on a smart phone (shown in Figure 7b). It is evident that paper-based cell analysis offers a new perspective on simplifying existing cell research methods as well as providing a wider prospect for developing paper chips.

### 5.4. Other Applications

Paper-based microfluidic chips can not only be directly applied to analyze and detect various biomarkers, but they can also help with other types of analyses. Samae et al. [106] used paraffin-treated paper to accurately prepare multilayer paper-based passive microfluidic micromixers. Comparatively, its zig-zag channel structure was studied for efficient mixing, enabling it to be docked with a variety of experimental equipment to play its role. Hong et al. [107] used photolithography to prepare the concentration gradient generator on the paper-based microfluidic chip. They successfully separated five concentration gradients of adriamycin. Then, they added different concentrations of adriamycin to the three-dimensional cell culture arrays to observe the effect of drug toxicity on tumor cells. Son et al. [108] made a cation-selective membrane by using cellulose paper and prepared a paper-based ion concentration polarization device that can achieve selective preconcentration of human breast cancer marker MUC1 gene fragment and Danon disease marker LAMP-2 gene fragment. Saha et al. [109] outlined the method of developing paper-based blood/plasma separation equipment by using the pore characteristics of different kinds of papers without adding additional power. With its low cost, high efficiency, and development potential, this equipment has several advantages. Niu et al. [110] used the principle of rapid isoelectric focusing to separate proteins. In paper fluid channels, they saw proteins in complex matrices become concentrated and separated at the same time. Paper-based microfluidic chips can not only do the analysis and detection function but also be used together with other experimental equipment, thereby broadening their applicability.

## 6. Summary and Outlook

The microfluidic chips made from paper would have profound effects on physics, biotechnology, and medicine. Rapid progress in microfluidic chips over the past decade makes us optimistic that this ambition is within reach. Among microfluidic chips of various materials, paper-based microfluidic chips are distinct for their small size, light weight, low cost, no additional driving force, easy processing, and environmental friendliness. Moreover, the excellent performance of paper chips makes them widely applicable for the detection of biochemical markers, nucleic acid preparation, cell analysis, and a variety of auxiliary analyses. Such extensive laboratory evolution has been used to promote biomedical legends. The advancement of science and the popularity of related equipment have made the preparation of paper chips a mature technology. Interestingly, mass production based on computer pattern design has further reduced the preparation cost of paper chips [111,112].

Currently, paper chip research focuses on efficiency, accuracy, integration, and innovation. Rather than making paper chips that can only detect one substance and only have one channel, it is becoming more common to make multi-channel paper chips that can detect and distinguish several substances at the same time [113,114,115]. Additionally, qualitative detection is being replaced by quantitative detection. This non-invasive technique not only detects the existence of substances but also determines their content in the tested products through parameters such as the depth of color development, the area of color change, the intensity of fluorescence, the size of the current, and so on [116,117,118,119]. It is common for the detection of a single substance to decrease the lower limit of detection and to continuously improve the sensitivity of detection. In order to enable non-professional users to obtain accurate detection results, the combination of paper chips and electronic devices is also gaining popularity. With the proliferation of multi-function paper chips, broader applications are possible besides just reading and detecting [120,121,122]. In summary, although there are considerable challenges to overcome, we remain cautiously optimistic that paper-based microfluidic chips—which can predict and trace biomarkers from home-grown samples to deliver efficient devices with desired functions—will eventually become a reality.

## Figures and Tables

**Table 1:** Materials of paper-based microfluidic chips [12,13,14].

Material	Characteristics	Applications
Filter paper	Suitable water absorption, easy to obtain, low cost, poor strength	Suitable for all kinds of paper chips, the most widely used paper-based materials
Nitrocellulose paper	It can bind and fix protein, and high cost	Detection based on Western blot reaction, colloidal gold test paper reaction zone
Glass fiber paper	Stable properties, not easy to break, high temperature resistance, corrosion resistance	Detection based on chemical reactions

**Table 2:** Preparation method of paper-based microfluidic chips.

Methods	Advantages	Disadvantages
Photolithography	The earliest method for making paper chips, precise channel structure [20]	The process is complicated The resulting paper chips are not suitable for bending [8]
Plasma treatment technology	More suitable for mass production and have low cost [21]	Depends on templates, reducing flexibility [22]
Wax printing	Simple processing, environmentally friendly materials [23,24]	Rely on wax spray printers, heating-induced horizontal diffusion reduces structure accuracy [25]
Inkjet method	Simple processing can be drawn with ink pen, no heating diffusion, more precise structure [26,27]	Hydrophobic inks can be toxic, ink pens are inaccurate for hand drawing, still rely on inkjet printers [28]
Screen printing	Ideal for mass production, simple process, and low cost [29,30]	Rely on templates, greatly reducing flexibility during research [31]
Laser processing technology	Very precise structures can be prepared [32]	Rely on expensive laser equipment and difficult to popularize [33]
3D origami method	3D structure has more functions, direct registration of each layer [34,35]	Means of fixing are required between layers, only single material can be used [36]
3D lamination method	3D structure has more functions, can use a variety of materials [37]	Fixed means are required between layers, registration methods are required [38]
Other 3D methods	Highly innovative and has huge development potential [39]	Special uses, difficult to promote [40]

**Table 3:** Analysis Method of paper-based microfluidic chips.

Methods	Advantages	Disadvantages
Colorimetric method	Intuitive results, easy to read with the naked eye, low cost [51,52]	Unable to achieve accurate quantitative detection [53]
Electrochemical method	Quantitatively accurate, fast reading [54,55]	Rely on electrochemical workstation, increase cost and reduce flexibility [56]
Fluorescence method	Low detection limit, very sensitive [57,58]	Relying on fluorescence detection equipment, easily affected by the signal of paper fluorescent agent [59]
United electronics	Combining the aforementioned methods enables non-professionals to obtain accurate results [60,61]	Need to install a mobile APP or even larger devices, reducing flexibility [4]

## Data Availability

Not applicable.
